# Characterization of Quality Components and Near-Infrared Spectroscopy Prediction Modeling for Free Amino Acids in Chongqing White Tea Based on Chemometrics

**DOI:** 10.3390/foods15213977

**Published:** 2026-11-05

**Authors:** Ying Zhang, Jie Wang, Ruiqi Duan, Rui Chang, Hongyu Luo, Juan Yang, Yingfu Zhong

**Affiliations:** Chongqing Academy of Agricultural Sciences, Chongqing 402160, China; bellazy@126.com (Y.Z.);

**Keywords:** Chongqing white tea, free amino acids, near-infrared spectroscopy, artificial neural network, digital model

## Abstract

To achieve rapid, non-destructive, and accurate digital evaluation of core quality components in Chongqing white tea, samples manufactured by multiple tea enterprises were investigated. Wet chemical analytical methods were conducted to determine primary biochemical components, followed by principal component analysis (PCA) and correlation analysis to identify the core quality-characterizing indicators. Near-infrared spectroscopy (NIRS) coupled with back-propagation artificial neural network (BP-ANN) models incorporating three transfer functions (linear [−1, 1], logistic, and tanh) were subsequently developed, and independent external samples were used to validate model generalization. The results indicated that the cumulative variance contribution rate of the first five principal components of biochemical components reached 90.957%. Ranked by their contribution to white tea sensory quality, free amino acids (AAs) served as the pivotal quality-determining indicator (r = 0.53, *p* < 0.05). Multiplicative scatter correction combined with first derivative (MSC + FD) was identified as the optimal spectral preprocessing method. Synergy interval partial least squares (si-PLS) screened four characteristic spectral regions: 4377.6–4751.7 cm^−1^, 4755.6–5129.7 cm^−1^, 6262.7–6633.9 cm^−1^, and 7386.0–7756.3 cm^−1^. The cumulative contribution rate of the first three principal components derived from the selected characteristic spectra reached 97.16%. The BP-ANN model utilizing the tanh transfer function achieved optimal predictive performance (Rp^2^ = 0.961, RMSEP = 0.253%, RPD = 4.336) and showed reliable practical generalization capability on external validation samples (Rp^2^ = 0.978, RMSEP = 0.231%). This study realizes rapid, non-destructive, and digitized quantitative determination of free amino acid content in Chongqing white tea, providing robust technical support for intelligent quality control in the white tea industry.

## 1. Introduction

Tea is one of the three major non-alcoholic beverages worldwide and is honored as the national beverage in China [1]. Chongqing white tea represents a distinctive premium green tea that has undergone rapid development in Chongqing Municipality in recent years. It is manufactured from early-spring tender shoots comprising one bud and one leaf from the tea cultivar *Camellia sinensis* cv. ‘Baiye 1’ through specialized processing steps, including spreading, fixation, shaping, and drying. Its core cultivation areas are situated in mountainous terrains at altitudes ranging from 600 to 1000 m, characterized by a warm and humid climate, persistent cloud and mist cover, and abundant diffuse light and short-wave ultraviolet radiation. This unique mountainous agro-ecological environment not only maintains the tenderness of the tea shoots, but also facilitates the active accumulation of favorable biochemical constituents, such as amino acids, proteins, and alkaloids, thereby establishing the exceptional sensory quality of Chongqing white tea. The finished tea exhibits straight and plump dry leaf appearance, vibrant tender-green color, clear and bright liquor, delicate and enduring aroma, and a refreshing, mellow, and sweet taste, enjoying extensive consumer popularity due to its prominent flavor attributes. Because tea sensory quality is the direct phenotypic manifestation of its internal physicochemical profile, systematically analyzing the biochemical characteristics of Chongqing white tea is of fundamental research significance for elucidating the material foundation underlying its quality formation and promoting the high-quality development of the tea industry.

At present, quantitative analysis of biochemical components in tea primarily relies on conventional wet chemical approaches. These methods mainly utilize analytical instruments such as high-performance liquid chromatography (HPLC) [2], gas chromatography (GC) [3], liquid chromatography–mass spectrometry (LC-MS) [4], and gas chromatography–mass spectrometry (GC-MS) [5] to quantify various chemical components, thereby achieving objective and accurate quality assessment. While traditional wet chemical methods offer high detection accuracy and standard reproducibility, acting as gold-standard reference methods in tea physicochemical detection, they present considerable drawbacks in practical implementations. These protocols involve laborious analytical procedures and rigorous sample pretreatment steps, requiring heavy manual workloads, excessive reagent and consumable consumption, and substantial operational costs. Moreover, they are time-consuming and inherently destructive, making them incapable of satisfying the urgent industrial demand for rapid, high-throughput, and on-line inspection of core quality constituents during the scale-up processing of Chongqing white tea. Consequently, establishing an efficient, convenient, cost-effective, and non-destructive quantitative technique is of vital practical importance for realizing the rapid digital screening of essential quality markers and refining the objective quality evaluation framework of Chongqing white tea.

In recent years, various rapid and non-destructive detection technologies have been widely promoted and applied in the quality evaluation of agricultural products, including hyperspectral imaging (HSI) [6], electrochemical detection [7], colorimetric sensor array (olfactory visualization) technology [8], and fluorescent probe techniques [9]. Although these technologies possess distinct advantages, they exhibit prominent limitations in large-scale tea screening scenarios. Hyperspectral imaging suffers from severe spatial and spectral redundancy, high hardware acquisition costs, and heavy computational burdens. Electrochemical sensing and fluorescent probe techniques offer remarkable sensitivity, yet they still require auxiliary chemical extraction, which is inherently destructive to samples and fails to achieve truly non-destructive analysis. In contrast, near-infrared spectroscopy (NIRS) [10] can rapidly extract internal chemical component information by probing the overtone and combination vibrational absorptions of hydrogen-containing bonds (such as O–H, C–H, and N–H). Attributed to its distinct advantages of non-destructiveness, high efficiency, simple operation, feasibility for online monitoring, and low operational costs, NIRS has been extensively applied in quality analysis across diverse agricultural commodities [11,12]. Currently, NIRS has been successfully implemented in numerous tea research areas, including overall quality assessment [13], accurate grade classification [14], and geographical origin tracing [15], demonstrating high technical maturity and broad application prospects. Nevertheless, NIR spectroscopy also has inherent technical limitations. As an indirect analytical technique, its prediction performance heavily depends on high-quality calibration datasets with sufficient sample representativeness. Model robustness can be easily disturbed by variations in sample particle size, moisture content, and matrix background. Moreover, it is relatively challenging to achieve high-accuracy prediction for low-concentration constituents, and established calibration models require regular updates with new samples to maintain stable generalization performance in practical applications [16].

Existing studies on Chongqing white tea have primarily focused on storage year identification [17], volatile aroma profiling [18], and biochemical variations during the withering process [19]. However, systematic research on the rapid prediction of key biochemical components in this distinctive tea category using NIRS remains scarce, hindering the industrial and intelligent demands for rapid quality control. Calibration algorithms are the cornerstone of quantitative NIRS analysis, directly governing the predictive accuracy and robustness of models. Partial least squares regression (PLSR) is a widely adopted classic linear algorithm that effectively resolves multicollinearity among spectral variables. In contrast, artificial neural networks (ANNs) possess superior non-linear mapping capabilities and can precisely capture the non-linear relationships between spectral signatures and physicochemical parameters in complex matrix systems, providing an effective technological pathway for high-precision tea quality modeling [20]. Therefore, taking Chongqing white tea as the research object, this study quantified the primary biochemical constituents using standard wet chemical methods and employed principal component analysis (PCA) and correlation analysis to identify the core quality-characterizing indicators and elucidate their intrinsic interrelationships. Furthermore, utilizing NIRS coupled with diverse chemometric algorithms—including synergy interval partial least squares (si-PLS), PCA, PLSR, and a back-propagation artificial neural network (BP-ANN)—high-precision quantitative prediction models for pivotal quality components were constructed. This study aims to establish a rapid, non-destructive, and accurate quality evaluation framework tailored for Chongqing white tea, providing robust technical and theoretical support for intelligent quality inspection and industrial quality control.

## 2. Materials and Methods

### 2.1. Materials

A total of 150 Chongqing white tea samples were investigated in this study. Fresh tea leaves for all samples were harvested strictly in accordance with the uniform standard of one bud and one leaf between 15 March 2025, and 10 April 2025. Samples were collected from four representative local manufacturers—Chongqing Shangming Agricultural Development Co., Ltd., Chongqing Fengjie Jiupin Tea Co., Ltd., Chongqing Fuling Zhinan Tea Factory, and Chongqing Yujiang Agricultural Development Co., Ltd.—covering the primary commercial production and processing entities to ensure sample representativeness. Using the Kennard–Stone (KS) algorithm, 120 samples designated for modeling were divided at a 3:1 ratio into a calibration set (*n* = 90) and a prediction set (*n* = 30). The calibration set was employed for model training and hyperparameter optimization, while the prediction set was utilized to evaluate goodness-of-fit and model robustness. The remaining 30 independent samples, excluded from the model development phase, served as an external test set to verify the practical generalization capability and external predictive accuracy of the models.

### 2.2. Instruments and Equipment

The primary instruments and equipment utilized in this experiment included: an HHS constant-temperature water bath (Shanghai Boxun Medical Biological Instrument Corp., Shanghai, China); UV-2550 UV–Vis spectrophotometer (Shimadzu Corp., Kyoto, Japan); Milli-RO PLUS 30 water purification system (Millipore S.A.S., Molsheim, France); Alliance 2695 high-performance liquid chromatography (HPLC) system coupled with a 2998 photodiode array detector (PDA) (Waters Corp., Milford, MA, USA); Phenomenex ODS C_18_ reversed-phase column (250 mm × 4.6 mm, 5 μm; Phenomenex Inc., Torrance, CA, USA).

### 2.3. Methods

#### 2.3.1. Sensory Evaluation of Tea

Sensory quality evaluation of Chongqing white tea was performed in strict accordance with the Chinese National Standard GB/T 23776–2018 [21]. Specifically, 3.0 g of each tea sample, obtained via the quartering method, was accurately weighed and transferred into a 150 mL standard evaluation cup. Boiling distilled water (100 °C) was infused into the cup, which was immediately covered and brewed statically for 4 min. Following infusion, the tea liquor was sequentially decanted into a standard tea evaluation bowl and evaluated once cooled to an appropriate sensory temperature. An evaluation panel comprising five licensed senior professional tea evaluators (three males and two females) conducted the sensory assessment. Samples were comprehensively scored on a 100-point scale across five core quality attributes: dry leaf appearance, liquor color, aroma, taste, and infused leaf. A higher comprehensive score corresponded to superior overall tea quality. To ensure the reliability and standardization of the evaluation results, all panellists were required to maintain optimal health and strict hygiene conditions, and smoking was strictly prohibited throughout the evaluation process to eliminate external sensory interference.

#### 2.3.2. Determination of Biochemical Components

##### Determination of Water Extracts

The content of water extracts in tea samples was determined by oven-drying of the extraction residue [22]. Approximately 2.000 g of finely ground and homogenized tea sample was accurately weighed into a 500 mL Erlenmeyer flask, followed by the addition of 300 mL of boiling distilled water. The mixture was immediately transferred to a boiling water bath and extracted for 45 min, with thorough shaking every 10 min to ensure complete contact between the sample matrix and boiling water. Upon extraction completion, the suspension was immediately subjected to hot vacuum filtration. The Erlenmeyer flask and the residue on the filter paper were rinsed thoroughly in small portions with boiling distilled water (approximately 150 mL in total) until the washings were clear and free of residual solutes. The filter paper bearing the tea residue was then transferred into a pre-weighed dish and dried to constant weight in an electrothermal constant-temperature drying oven at 120 °C (initially dried for 1–2 h, cooled to ambient room temperature in a desiccator, weighed, and redried for 1 h until the difference between consecutive weighings did not exceed 0.001 g). In conjunction with the dry matter content of the sample, the dry weight of the residue was calculated by mass difference to determine the mass fraction of water extracts (%).

##### Determination of Tea Polyphenols

Tea polyphenol content was quantified using the Folin–Ciocalteu colorimetric method [23]. Aliquots (1.0 mL) of gallic acid standard working solutions, distilled water (blank), and prepared tea extract solutions were accurately transferred into separate 10 mL test tubes. To each tube, 5.0 mL of 10% (*v*/*v*) Folin–Ciocalteu reagent was added, vortexed thoroughly, and allowed to react at room temperature in the dark for 3–8 min. Subsequently, 4.0 mL of 7.5% (*w*/*v*) sodium carbonate solution was added, and the volume was adjusted to the 10 mL mark with deionized water. The reaction mixture was vortexed again and incubated in the dark at room temperature for 60 min. The absorbance was measured at 765 nm against the reagent blank using a UV–Vis spectrophotometer equipped with a 10 mm optical path length quartz cuvette. The mass fraction of tea polyphenols in the samples was calculated based on the standard calibration curve.

##### Determination of Free Amino Acids

The total free amino acid content was determined using the ninhydrin colorimetric method [24]. Aliquots (1.0 mL) of the tea extract solution, series gradient concentrations of theanine standard working solutions, and distilled water (reagent blank) were transferred into separate 25 mL stoppered colorimetric tubes. Subsequently, 0.5 mL of phosphate-buffered solution (pH 8.0) and 0.5 mL of 2% (*w*/*v*) ninhydrin solution were sequentially added. The mixture was shaken thoroughly and heated in a boiling water bath for 15 min. Upon completion of heating, the tubes were rapidly cooled to room temperature under running water, diluted to the 25 mL mark with deionized water, and mixed thoroughly by inversion. After incubating in the dark at room temperature for 10 min, the absorbance was recorded at 570 nm using a UV–Vis spectrophotometer equipped with a 5 mm optical path length quartz cuvette. The mass fraction of total free amino acids (%) in tea samples was calculated based on the theanine calibration curve.

##### Determination of Gallic Acid, Caffeine, and Catechin Monomers

The contents of gallic acid (GA), caffeine (CAF), and individual catechin monomers were quantified by high-performance liquid chromatography (HPLC) [25]. Approximately 0.2000 g of finely ground and homogenized sample was accurately weighed into a 50 mL stoppered test tube, followed by the addition of 10.0 mL of 70% (*v*/*v*) aqueous methanol preheated to 70 °C. The tube was sealed, mixed, and extracted in a water bath at 70 °C in the dark for 10 min, with vortex mixing every 5 min. After cooling to room temperature, the suspension was centrifuged at 3500 r/min for 10 min, and the supernatant was quantitatively decanted into a 10 mL volumetric flask. The remaining residue was re-extracted with 5.0 mL of preheated 70% methanol under identical conditions. After centrifugation, the supernatants were combined, diluted to the 10 mL mark with 70% methanol, thoroughly mixed, and filtered through a 0.45 μm organic microporous membrane. Subsequently, 2.0 mL of the filtrate was accurately diluted to 10 mL with deionized water in a volumetric flask for HPLC analysis.

Chromatographic separation was performed on an HPLC system equipped with a UV/diode array detector (UV/DAD) using a reversed-phase C_18_ column (250 mm × 4.6 mm, 5 μm). The mobile phase consisted of acetonitrile (phase A) and an aqueous solution containing 0.1% formic/acetic acid (phase B) under gradient elution. The column temperature was maintained at 35 °C, and the detection wavelength was monitored at 278 nm. Target compounds were identified by comparing retention times with authentic standards and quantified by the external standard peak area method to determine mass fractions (%) in the tea samples.

##### Determination of Tea Polysaccharides

Tea polysaccharide content was determined using the anthrone–sulfuric acid colorimetric method [26]. Approximately 1.000 g of ground and sieved tea powder was accurately weighed into a round-bottom flask, and 40 mL of 80% (*v*/*v*) aqueous ethanol was added. The mixture was refluxed in a 95 °C water bath for 1 h to eliminate lipids, pigments, free monosaccharides, and polyphenolic interferences. After cooling, the mixture was filtered under vacuum, and the residue was washed twice with 10 mL of 80% ethanol, followed by air-drying at room temperature to remove residual solvent. The dried residue and filter paper were returned to the flask, mixed with 100 mL of distilled water, and extracted in a boiling water bath (100 °C) for 1 h. The extract was filtered while hot under vacuum, and the residue was washed twice with 10 mL of hot distilled water. The combined filtrates were centrifuged at 4000 r/min for 10 min, and the supernatant was transferred into a 100 mL volumetric flask and made up to volume with distilled water. An aliquot of 1.0 mL of the sample extract (with 1.0 mL of distilled water as a blank) was pipetted into a stoppered test tube, and 4.0 mL of 0.2% (*w*/*v*) anthrone–concentrated sulfuric acid solution was slowly introduced along the tube wall. The solution was immediately vortexed and cooled in an ice bath. The tube was then incubated in a boiling water bath for exactly 7 min, rapidly cooled to room temperature, and maintained in the dark for 10 min. The absorbance was measured at 620 nm using a UV–Vis spectrophotometer with a 10 mm optical path length cuvette. The mass fraction of tea polysaccharides was calculated based on the glucose standard calibration curve.

### 2.4. Near-Infrared Spectroscopy and Chemometric Methods

#### 2.4.1. Near-Infrared Spectral Acquisition

Near-infrared spectra were acquired using an Antaris II Fourier-transform near-infrared (FT-NIR) spectrometer (Thermo Fisher Scientific Inc., Waltham, MA, USA) equipped with an indium gallium arsenide (InGaAs) detector. Spectral scanning was performed over the wavenumber range of 4000–10,000 cm^−1^ at a spectral resolution of 8 cm^−1^. Prior to spectral collection, the spectrometer was preheated for 1 h until reaching operational thermal equilibrium to minimize systematic instrumental drift. Prior to spectral acquisition, all tea samples were ground using a grinding mill and sieved through a 60-mesh sieve (250 μm). No additional drying or chemical pretreatment was performed. During measurement, ground tea samples were loaded uniformly into the matching quartz sample cup and spectra were collected in diffuse reflectance mode. To acquire representative spectral signals and eliminate optical path variations caused by uneven sample packing, the sample cup was continuously rotated through 360° at a constant speed during the acquisition process. Each sample was scanned in triplicate, and the mean spectrum was taken as the raw spectral data for subsequent preprocessing and chemometric modeling.

#### 2.4.2. Spectral Data Preprocessing and Feature Interval Selection

Following spectral acquisition, the raw spectra were digitized into 1557 data points per spectrum with a sampling interval of 3.86 cm^−1^ and exported to Microsoft Excel for storage. To mitigate baseline drift, light scattering interference, and random instrument noise, as well as to enhance spectral signal-to-noise ratio and data efficacy, nine preprocessing methods were systematically evaluated. These methods included standard normal variate (SNV), multiplicative scatter correction (MSC), first derivative (FD), second derivative (SD), and various combined preprocessing techniques. Based on the performance of partial least squares (PLS) regression models, the cross-validation determination coefficient (Rc^2^) and root mean square error of cross-validation (RMSECV) were designated as the primary evaluation criteria. Adhering to the optimization rule of maximizing Rc^2^ and minimizing RMSECV, the optimal spectral preprocessing strategy for Chongqing white tea was determined.

To further eliminate non-informative spectral redundancy and identify characteristic absorption bands associated with key quality components, synergy interval partial least squares (si-PLS) was implemented for spectral interval selection [27]. The preprocessed full spectra were equidistantly partitioned into 10–24 subintervals. Calibration PLS models were subsequently constructed by permuting combinations of 2–4 optimal subintervals. Guided by the minimum RMSECV criterion, the spectral regions exhibiting the highest correlation with essential quality indices of Chongqing white tea were isolated, effectively reducing model complexity and enhancing both predictive accuracy and robustness.

The calculation formula for RMSECV is expressed as follows:

(1) $$\mathrm{RMSECV}=\sqrt{\frac{\sum_{i=1}^{n}{\left(y_{i}^{\prime }-y_{i}\right)}^{2}}{n}}$$

where *n* represents the number of samples in the calibration set, *y_i_* is the reference measured value of sample *i*, and $y_{i}^{\prime }$ is the cross-validated predicted value of sample *i* in the calibration set.

#### 2.4.3. Calibration Modeling Methods

In this study, both linear and non-linear modeling algorithms were implemented to construct quantitative prediction models for the biochemical components of Chongqing white tea, specified as follows:

(1) Partial least squares regression (PLSR): PLSR is a multivariate statistical modeling approach that integrates the advantages of principal component analysis (PCA), canonical correlation analysis (CCA), and multiple linear regression (MLR). While compressing the dimensionality of high-dimensional spectral arrays, PLSR maximizes the covariance between the independent variables (spectral features) and the dependent variables (physicochemical parameters). This technique effectively overcomes multicollinearity and the “curse of dimensionality” inherent in continuous spectral data, exhibiting robust noise resistance and generalization performance; consequently, it has been widely and maturely applied in quantitative NIRS analysis [28].
(2) Back-propagation artificial neural network (BP-ANN): BP-ANN represents a quintessential non-linear machine learning framework. Its underlying mechanism involves calculating the error gradients between model-predicted values and reference values via the chain rule, followed by propagating the error backwards layer by layer from the output layer to the input layer. In conjunction with the gradient descent optimization algorithm, the connection weights and biases across network layers are iteratively updated to minimize the global objective loss function, thereby realizing accurate mapping and non-linear fitting between complex spectral features and target chemical indices [29].

#### 2.4.4. Model Evaluation

All spectral preprocessing, feature variable screening, and chemometric modeling were performed using MATLAB 2012a software (MathWorks, Natick, MA, USA). To objectively and quantitatively assess the goodness-of-fit, predictive precision, and generalization capability of the developed calibration models, the determination coefficient of cross-validation (Rc^2^), determination coefficient of prediction (Rp^2^), root mean square error of cross-validation (RMSECV), root mean square error of prediction (RMSEP), and residual predictive deviation (RPD) were employed as the core evaluation metrics [30]. Model evaluation followed unified assessment criteria: higher R^2^ and RPD values, concurrent with lower RMSECV and RMSEP values, indicate superior fitting accuracy, higher stability, and robust predictive capacity. All statistical evaluation indicators were computed in accordance with universal standard formulation protocols for near-infrared quantitative modeling.

RPD = SD/RMSEP(2)

(3) $$\mathrm{RMSEP}=\sqrt{\frac{\sum_{i=1}^{n}{\left(y_{i}-y_{i}^{\prime }\right)}^{2}}{n}}$$

(4) $$R^{2}=1-\frac{\sum_{i=1}^{n}{\left(y_{i}^{\prime }-y_{i}\right)}^{2}}{\sum_{i=1}^{n}{\left(y_{i}-\bar{y}\right)}^{2}}$$

where *SD* is the standard deviation of prediction set; *n* is the number of samples in the prediction set; *y_i_* denotes the actual value for sample *i*, and $y_{i}^{\prime }$ refers to the value for sample *i* in the prediction set; $\bar{y}$ is the mean true value across whole samples. Model predictive performance was categorized according to established RPD thresholds: an RPD ≥ 2.0 demonstrates excellent predictive reliability and quantitative applicability; 1.4 ≤ RPD ≤ 2.0 indicates acceptable rough estimation capability; and an RPD < 1.4 indicates that the model is unreliable for quantitative prediction [31].

### 2.5. Applied Statistical Analysis

All chemical composition data and sensory scores were subjected to descriptive statistics, Pearson correlation analysis and principal component analysis (PCA) [32]. The maximum, minimum, mean and standard deviation (SD) of all biochemical components and sensory scores in Table 1 were calculated to characterize the central tendency, variation range and inter-sample variability of each quality parameter. This descriptive overview reveals the homogeneity of sensory quality and the variation in biochemical composition among the Chongqing white tea samples, which lays a foundation for the subsequent correlation analysis and PCA to interpret the links between chemical constituents and sensory attributes. Pearson correlation analysis was used to reveal the associations among different quality and amino acid variables. PCA was further adopted to reduce the dimensionality of the multivariate chemical dataset. Notably, this PCA was performed exclusively on biochemical variables, and sensory scores were not included in the PCA dataset. The analysis aimed to identify major sources of variation, visualize sample grouping patterns, and interpret the intrinsic relationships among these chemical variables. Compared with other unsupervised methods such as hierarchical cluster analysis (HCA), PCA provides continuous principal component scores for intuitive ordination of samples and straightforward interpretation of variable loadings, which is suitable for exploring the overall variation structure of the multiple chemical and amino acid parameters in this study.

**Figure 1:**
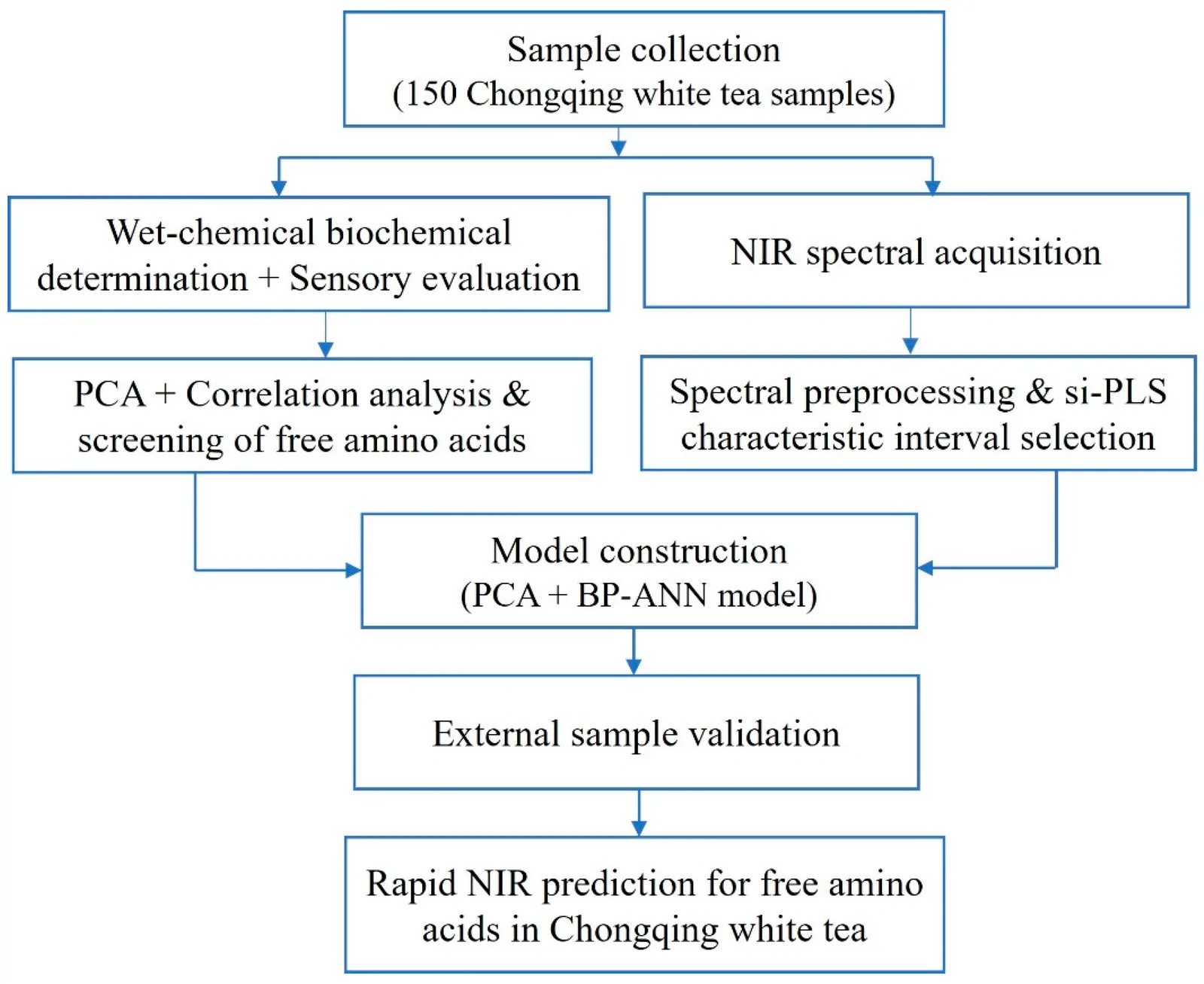
Schematic workflow diagram of the present study. Note: PCA: principal component analysis; si-PLS: synergy interval partial least squares; BP-ANN: back-propagation artificial neural network.

All statistical analyses were performed using Origin 2024b and R 4.2.2 software. The overall experimental workflow of this study is summarized in Figure 1.

## 3. Results and Discussion

### 3.1. Sensory Quality Scores and Biochemical Component Profiles

Sensory quality scores of Chongqing white tea samples were evaluated by professional tea tasting panelists, and primary biochemical components were quantitatively determined using standard analytical methods. The descriptive statistical results of sensory quality scores and chemical constituents are summarized in Table 1.

As shown in Table 1, the sensory quality scores of the Chongqing white tea samples ranged from 87.60 to 95.70 points, with an average score of 90.72 points and a standard deviation (SD) of only 1.51 points. This indicates that the collected tea samples exhibited high overall homogeneity, excellent stability, and strong batch consistency. Regarding the biochemical constituents, noticeable variations were observed among different physicochemical parameters, revealing distinct compositional characteristics.

Among all measured components, water extracts showed the highest average content at 44.25%, serving as the essential material foundation for the concentration, richness, and body of Chongqing white tea liquor. These were followed by total catechins, free amino acids, tea polyphenols, and caffeine, with average contents of 10.64%, 6.56%, 5.68%, and 3.18%, respectively, which collectively dictate the flavor profile of Chongqing white tea. Notably, the free amino acid content remained at a prominently elevated level (averaging 6.56%), acting as the crucial biochemical basis for the refreshing, mellow, sweet, and low-astringency taste of Chongqing white tea, which aligns closely with its distinct sensory characteristics. In contrast, individual monomeric components such as gallic acid (GA), gallocatechin (GC), and catechin (C) displayed relatively low concentrations, with average contents below 0.5%. Nevertheless, the absolute concentration of chemical constituents in tea is relative; while these components all contribute to quality formation, their specific degrees of influence differ substantially. To identify the core constituents with pivotal contributions to the overall quality of Chongqing white tea, principal component analysis (PCA) and correlation analysis were further performed.

### 3.2. Principal Component Analysis and Correlation Analysis

#### 3.2.1. Principal Component Analysis of Biochemical Components

As indicated in Table 2, PC1 primarily integrated information from catechin compounds, including catechin (C), epigallocatechin (EGC), and epigallocatechin gallate (EGCG). These secondary metabolites represent the principal contributors to the bitterness and astringency of tea infusions. In particular, ester-type catechins such as EGCG exert a dominant effect on taste briskness, astringency, and thickness; consequently, PC1 was defined as the “bitterness and astringency factor”. PC2 primarily characterized the variations in free amino acids (AAs), caffeine (CAF), and tea polyphenols (TPs). Amino acids serve as the central substance governing the umami and brisk taste of tea liquor, caffeine imparts thickness, body, and brisk bitterness, whereas tea polyphenols are the major source of astringency. A higher score along PC2 signifies abundant fresh-tasting constituents coupled with comparatively low levels of astringent compounds, imparting a fresh, brisk, and mellow character to the infusion. Hence, PC2 was designated as the “freshness and mellowness factor”. PC3 predominantly captured the variation in non-ester catechins—such as gallocatechin (GC), epigallocatechin (EGC), and epicatechin (EC)—as well as total polysaccharides (TS), while the ester-type catechin EGCG exhibited a negative loading. Non-ester catechins possess mild astringency and lower sensory pungency, whereas soluble polysaccharides enhance liquor viscosity, smoothness, and sweet aftertaste; therefore, PC3 was characterized as the “mellow and sweet-aftertaste factor”. PC4 comprehensively integrated the total contents of major constituents, including total catechins (TC), tea polyphenols (TPs), and total polysaccharides (TS). These bulk components collectively determine the concentration, richness, and depth of the tea infusion, serving as the material foundation for taste strength; thus, PC4 was defined as the “richness and thickness factor”.

**Figure 2:**
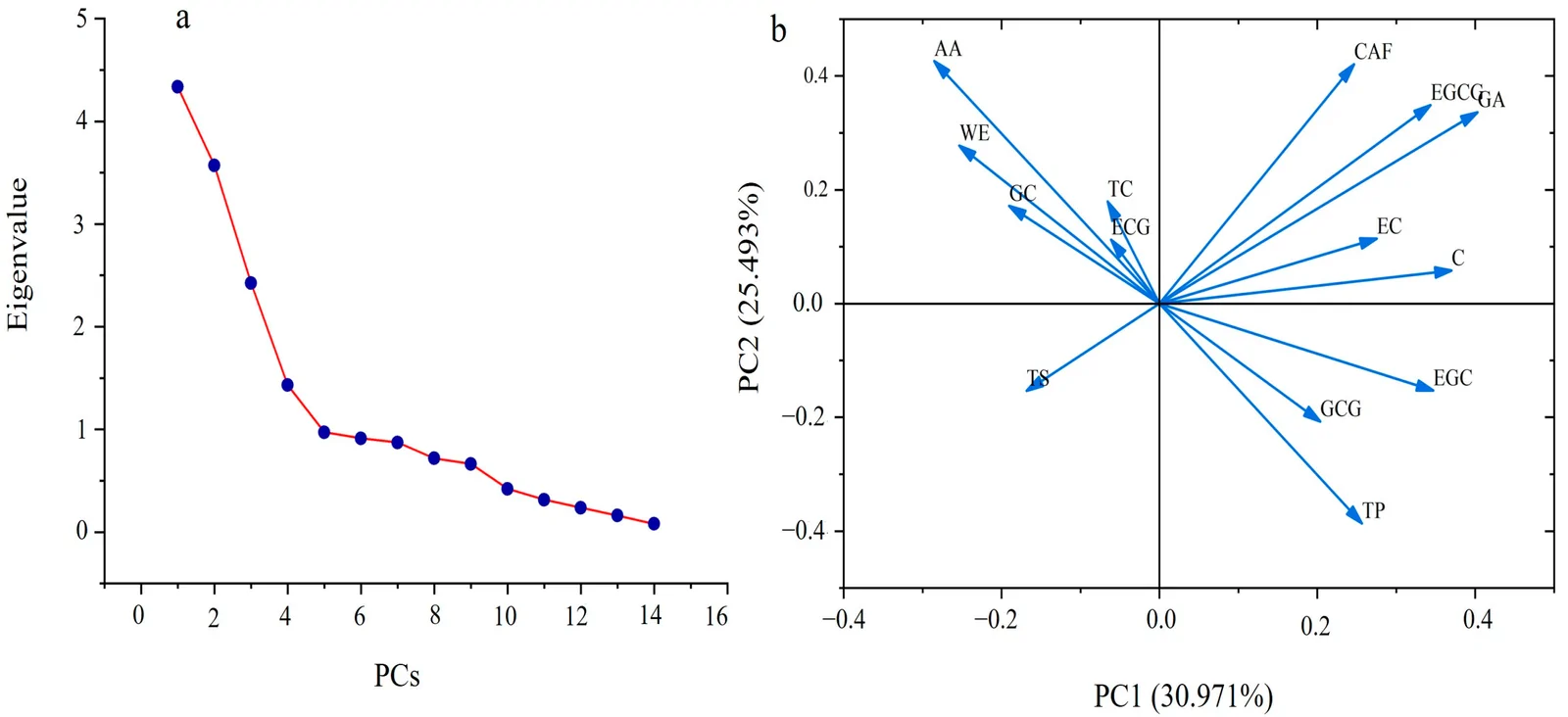
PCA of biochemical components in Chongqing white tea. (**a**) PCA scree plot; (**b**) PCA loading plot.

Table 2 and Figure 2a jointly demonstrated that the cumulative variance contribution rate of the first five principal components reached 90.957%, with the first four components exhibiting eigenvalues greater than 1.0 and accounting for 84.014% of the total variance. According to Kaiser’s criterion in principal component analysis [30], the first four principal components were sufficient to explain the vast majority of the variance in the biochemical profiles of Chongqing white tea. Specifically, PC1 and PC2 accounted for 30.971% and 25.493% of the total variance, respectively, yielding a combined variance explanation of 56.464%, and thus represented the primary axes driving the taste quality differences among samples. The PCA loading plot (Figure 2b) illustrated the correlations between individual physicochemical parameters and the principal components, as well as the intrinsic interrelationships among variables. Regarding projections along the PC1 axis, indices such as GA, C, EGCG, EGC, CAF, EC, GCG, and TP exhibited positive loadings, indicating positive correlations with PC1. Along the PC2 axis, AA, WE, GC, TC, ECG, GA, EGCG, CAF, and EC showed positive loadings and correlated positively with PC2. The angles between loading vectors directly reflected the degree of inter-variable correlation: the acute angles between EGCG and GA, as well as between WEs and AAs, indicated strong positive correlations; conversely, the vectors of AAs and TPs were oriented in nearly opposite directions (approaching 180°), demonstrating a prominent negative correlation. Furthermore, vector length signified the contribution of each variable to sample discrimination: AA, WE, CAF, TP, GA, and EGCG displayed longer vectors, identifying them as the dominant variables driving sample divergence in the PC1–PC2 coordinate plane. In contrast, ECG and TC had relatively short vectors, indicating that their contributions in this two-dimensional projection were limited and that their variance information was largely retained in higher-order components (PC3–PC5).

Collectively, principal component analysis effectively uncoupled the core biochemical components governing white tea quality, mapped them onto specific sensory dimensions, and established a reliable framework for subsequent correlation analysis and precision screening of essential quality markers.

#### 3.2.2. Correlation Analysis of Biochemical Components in Chongqing White Tea

**Figure 3:**
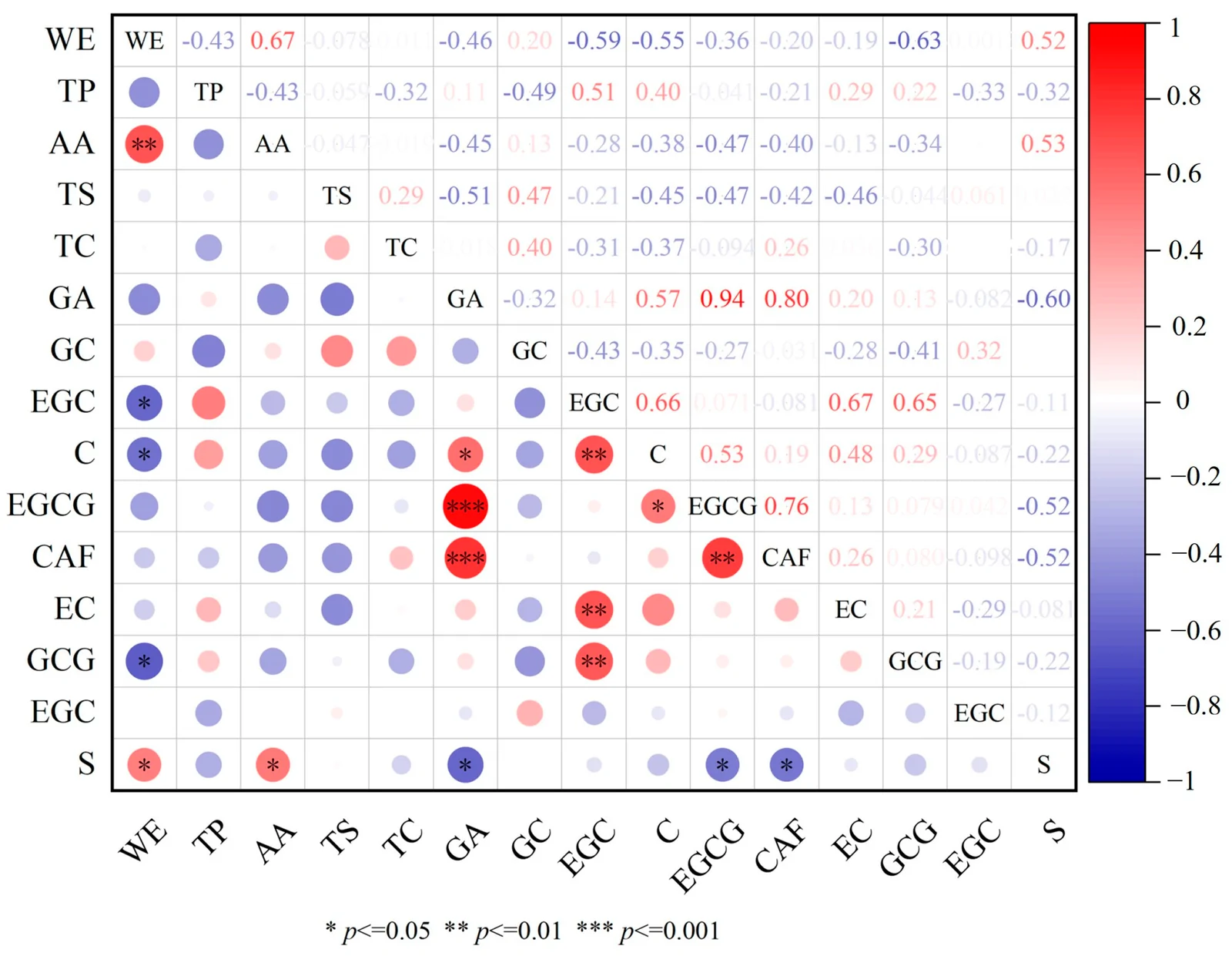
Correlation analysis among biochemical components and sensory scores. Note: WE: water extract; TPs: tea polyphenol; AAs: amino acid; TS: total sugar; TC: total catechins; GA: gallic acid; GC: gallocatechin; EGC: epigallocatechin; C: catechin; EGCG: epigallocatechin gallate; CAF: caffeine; EC: epicatechin; GCG: gallocatechin gallate; ECG: epicatechin gallate; S: score.

To elucidate the intrinsic metabolic interrelationships among biochemical components, investigate the synergistic and antagonistic dynamics during the accumulation of flavor compounds, and precisely identify core indicators tightly coupled with sensory quality based on PCA, correlation analysis was performed on the quality parameters of the tea samples (Figure 3).

Figure 3 shows that the sensory quality score (S) was significantly positively correlated with water extracts (WEs, r = 0.52, *p* < 0.05) and free amino acids (AA, r = 0.53, *p* < 0.05). Water extracts encompass an array of taste-active compounds including polyphenols, amino acids, and soluble sugars, serving as an integrative measure of the richness, thickness, and body of tea liquor; higher concentrations render the infusion more mellow and full-bodied [33]. Free amino acids are the pivotal contributors to the brisk, fresh, mellow taste and delicate aroma of white tea [34]. The coordinated accumulation of both constituents exerts a positive determining effect on overall sensory quality. Conversely, S showed a highly significant negative correlation with gallic acid (GA, r = −0.60, *p* < 0.05), alongside significant negative correlations with epigallocatechin gallate (EGCG, r = −0.52, *p* < 0.05) and caffeine (CAF, r = −0.52, *p* < 0.05). EGCG represents the dominant ester-type catechin, which, together with caffeine, forms the primary biochemical basis of bitterness and astringency. The accumulation of free GA often coincides with the hydrolysis of galloylated catechins or excessive structural conversions during processing. When bitter and astringent constituents accumulate excessively, they mask the fresh-sweet character of the infusion and disrupt flavor balance, thereby diminishing sensory scores. Other constituents—such as total soluble sugars (TS, r = −0.062), total catechins (TC, r = −0.28), and epicatechin (EC)—did not exhibit statistically significant correlations with quality scores (*p* > 0.05). Nevertheless, they displayed complex dynamic interactions, demonstrating that white tea quality is governed by an integrated balance of diverse taste compounds rather than the isolated concentration of a single component.

Furthermore, AAs were significantly positively correlated with WEs (r = 0.67, *p* < 0.01), but negatively correlated with tea polyphenols (TPs, r = −0.43). This trend reflects the classic trade-off between carbon and nitrogen metabolic fluxes in high-grade white teas, where elevated amino acid levels correspond to a lower polyphenol-to-amino acid (TP/AA) ratio—the biochemical prerequisite for the signature “fresh, mellow, sweet, and non-astringent” flavor. Extremely strong positive synergistic correlations were observed for GA with EGCG (r = 0.94, *p* < 0.001) and CAF (r = 0.80, *p* < 0.001). Similarly, tight synchronous variations were documented between EGCG and CAF (r = 0.76, *p* < 0.01), as well as between EGCG and catechin (C, r = 0.53, *p* < 0.05). These correlations indicate that ester-type catechins and purine alkaloids undergo concerted co-accumulation or participate in complexation within Chongqing white tea.

In summary, integrating the results of PCA and correlation analysis confirmed that the primary physicochemical indicators governing Chongqing white tea quality comprise free amino acids, water extracts, gallic acid, EGCG, and caffeine. Among these, free amino acids exhibited the strongest association with sensory scores. This biochemical profile aligns with the “refreshing, mellow, and sweet” sensory attributes of Chongqing white tea, highlighting its amino-acid-rich characteristics. These findings streamline the necessary analytical indicators, reduce redundant laboratory workloads, and provide a solid biochemical foundation for subsequent high-throughput non-destructive detection and digitized quality control.

### 3.3. Construction of NIRS Models for the Key Component Aa

#### 3.3.1. Comparison of Spectral Data Preprocessing Methods

**Figure 4:**
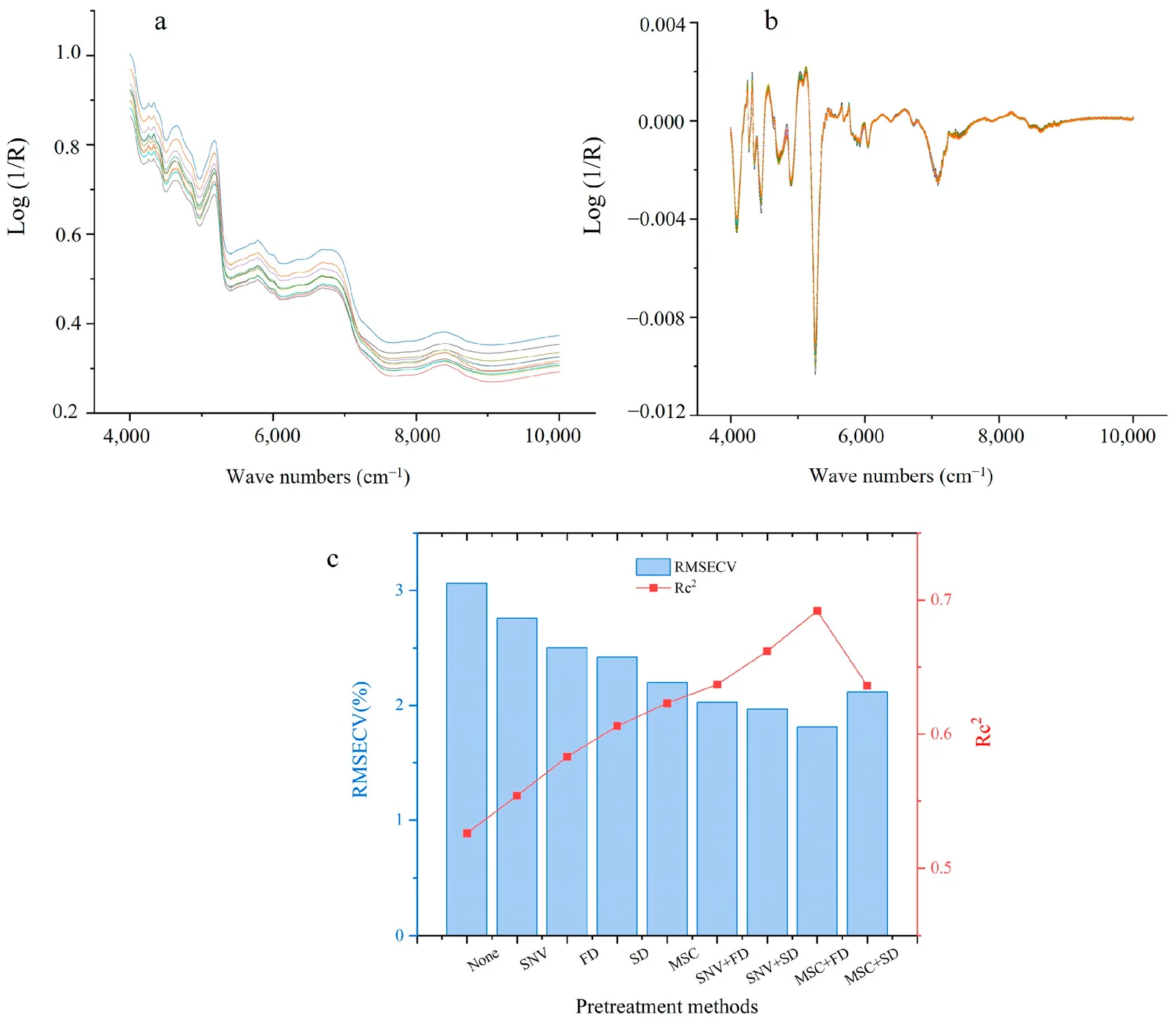
Comparison of PLS model performance under different spectral preprocessing methods. (**a**) Raw near-infrared spectra of white tea; (**b**) MSC + FD preprocessed spectra of white tea; (**c**) Comparison of results from nine spectral preprocessing methods. Note: Rc^2^: coefficient of determination of cross-validation; RMSECV, root mean square error of cross-validation; SNV: standard normal variate; MSC: multiple scatter correction; FD: first-order derivative; SD: second-order derivative.

As a critical biochemical indicator governing the sensory quality of Chongqing white tea, the rapid and non-destructive quantification of free amino acids (AAs) is of great practical significance. As illustrated in Figure 4a, the near-infrared spectra exhibited distinct absorption peaks within the wavenumber region of 4000–7000 cm^−1^, where absorbance intensities were intrinsically associated with the contents of constituent chemical compounds. However, because the absolute content of AAs was relatively low and susceptible to interference from the strong overtone and combination vibrations of O–H bonds in water, its characteristic absorption peaks were prone to being overlapped and masked by other major biochemical components. This spectral interference inevitably degrades the predictive capability of NIRS models. Consequently, prior to quantitative modeling, spectral preprocessing was essential to eliminate background noise, correct baseline shifts, and enhance the spectral signal-to-noise ratio (SNR). In this study, the effects of nine distinct preprocessing algorithms on the performance of partial least squares (PLS) models were systematically evaluated, as depicted in Figure 4c.

As demonstrated in Figure 4c, the model established using raw spectra without preprocessing yielded the poorest performance (Rc^2^ = 0.526, RMSECV = 3.065%), indicating severe noise interference in the collected raw spectra. Among the single preprocessing methods, SD achieved the most notable improvement (Rc^2^ = 0.623, RMSECV = 2.197%), followed by FD and MSC, all of which enhanced model performance to varying degrees. In comparison, combined preprocessing techniques generally outperformed individual algorithms. In particular, the combination of multiplicative scatter correction and first derivative (MSC + FD) produced the most substantial enhancement, elevating Rc^2^ to 0.692 and lowering RMSECV to 1.814%. This improvement demonstrates that MSC effectively eliminated light scattering interference caused by uneven particle spacing and sample voids, while the first derivative resolved overlapping absorption bands and corrected baseline drift, thereby enhancing spectral resolution and accentuating the characteristic spectral information of amino acids in white tea—findings that align with previously reported observations [35]. Consequently, implementing spectral preprocessing prior to calibration model development is essential, and MSC + FD was identified as the optimal preprocessing strategy, serving as the standardized preliminary step for all subsequent modeling procedures (Figure 4b). Nevertheless, despite the performance gains achieved through preprocessing optimization, the predictive accuracy remained insufficient for precise quantitative determination of AAs in white tea, necessitating characteristic wavelength selection techniques to eliminate uninformative and redundant spectral variables.

#### 3.3.2. Selection of Characteristic Spectral Regions Based on Si-Pls

Although spectral preprocessing effectively eliminated extraneous background noise, near-infrared spectral profiles remain complex due to severe peak overlap, baseline interference, and cross-coupling effects across diverse functional groups. Therefore, selecting spectral subintervals closely associated with amino acid constituents was essential to improve the predictive accuracy and parsimony of the calibration model. In this study, synergy interval partial least squares (si-PLS) was implemented for feature variable selection. The full spectrum (4000–10,000 cm^−1^) was split into 12–25 subintervals, and PLS models were developed based on all possible combinations of 2–4 intervals (Table 3). The optimal spectral subintervals were identified when the minimum RMSECV was reached, which reduced spectral dimensionality, simplified model complexity, and reinforced model robustness.

As shown in Table 3, the model performance exhibited noticeable fluctuations with varying numbers of partitioned subintervals. As the number of subintervals increased, the RMSECV of the developed PLS models initially decreased and subsequently increased. The model achieved optimal performance when the full spectrum was divided into 16 subintervals with the combination of four intervals [2 3 7 10]. Under this optimal condition, Rc^2^ reached 0.812 and RMSECV decreased to 1.225%, corresponding to the wavenumber ranges of 4377.6–4751.7 cm^−1^, 4755.6–5129.7 cm^−1^, 6262.7–6633.9 cm^−1^, and 7386.0–7756.3 cm^−1^. These results demonstrate that the si-PLS algorithm effectively isolated and concentrated on the spectral regions most closely related to amino acid contents in white tea samples. Although the selected characteristic spectral intervals accounted for only 25% of the full spectral variables, the predictive capability of the calibration model was further improved [36]. From a chemometric and vibrational perspective, the region of 4377.6–5129.7 cm^−1^ corresponds to the first overtone absorption of C–H bonds and the second overtone absorption of C=O bonds; the region of 6262.7–6633.9 cm^−1^ is assigned to the second overtone absorption of N–H bonds; and the region of 7386.0–7756.3 cm^−1^ is attributed to the second overtone absorption of C–H bonds [37]. These characteristic functional groups represent the principal chemical bonds associated with amino acids in near-infrared spectroscopy. The extraction of these absorption bands by the si-PLS method effectively captured the intrinsic spectral information of amino acids in Chongqing white tea.

#### 3.3.3. Principal Component Analysis of Characteristic Spectra

**Figure 5:**
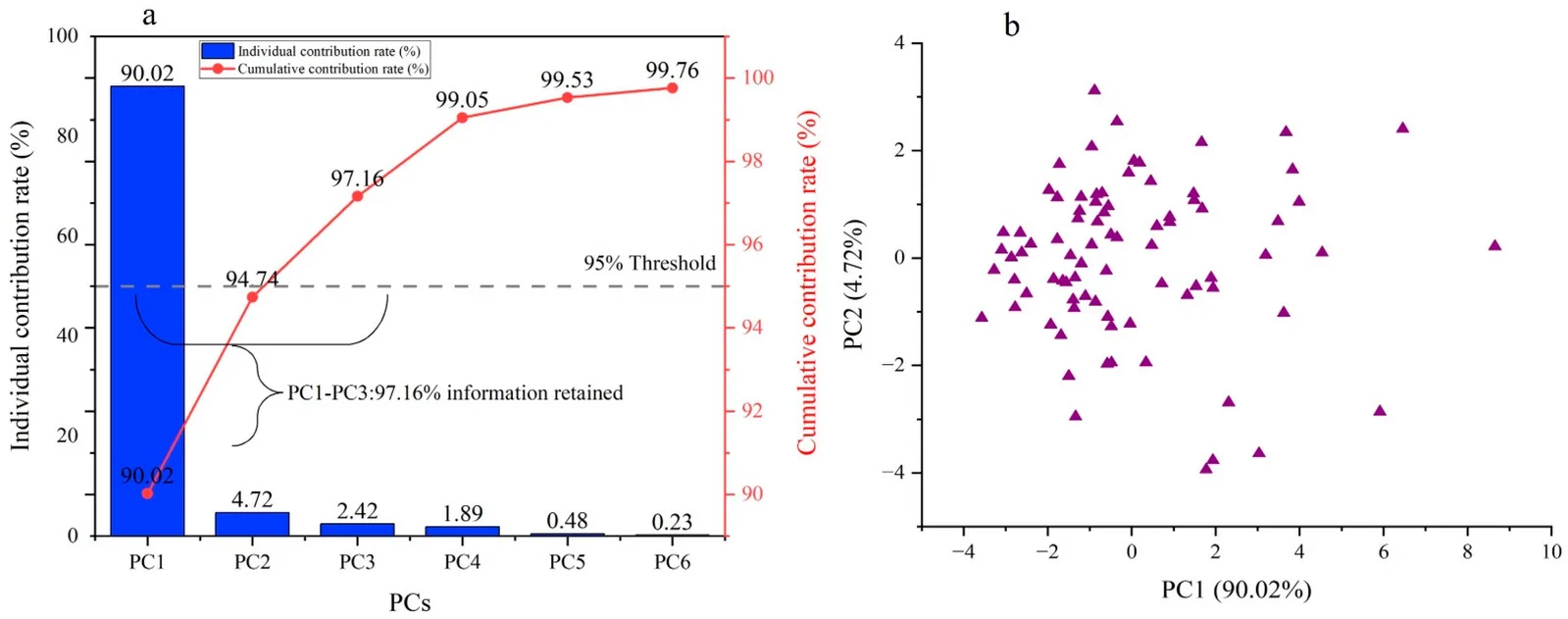
PCA results of the spectral dataset. (**a**) Variance contribution rates of the first six principal components extracted from the characteristic spectra; (**b**) PC1 vs. PC2 score plot.

Prior to developing the back-propagation artificial neural network (BP-ANN) prediction model for free amino acids (AAs), reducing the dimensionality of input spectral variables was required to avoid overfitting and network complexity. Therefore, principal component analysis (PCA) was conducted on the characteristic spectral intervals selected by si-PLS, as illustrated in Figure 5.

As depicted in Figure 5a, following PCA decomposition of the selected characteristic spectra, the individual variance contribution rate decreased sharply with the increasing number of principal components. Specifically, PC1 alone accounted for 90.02% of the total variance, and the cumulative variance contribution rate of the first six principal components (PC1–PC6) reached 99.76%. Notably, the first three principal components (PC1–PC3) accounted for a cumulative variance contribution rate of 97.16%, surpassing the conventional 95% information retention threshold. As shown in Figure 5b, most samples cluster in the score space, with a small number distributed on the positive side of PC1. No obvious outliers are detected, revealing that spectral variations between samples are dominated along the PC1 axis. This stable spectral data structure provides a reliable basis for the subsequent development of the BP-ANN model. According to the foundational principles of principal component analysis [30], the score vectors of the first three principal components adequately captured virtually all the essential spectral features contained within the screened characteristic intervals, providing an optimal, low-dimensional input vector for subsequent neural network training.

#### 3.3.4. Development of Artificial Neural Network Prediction Models

**Figure 6:**
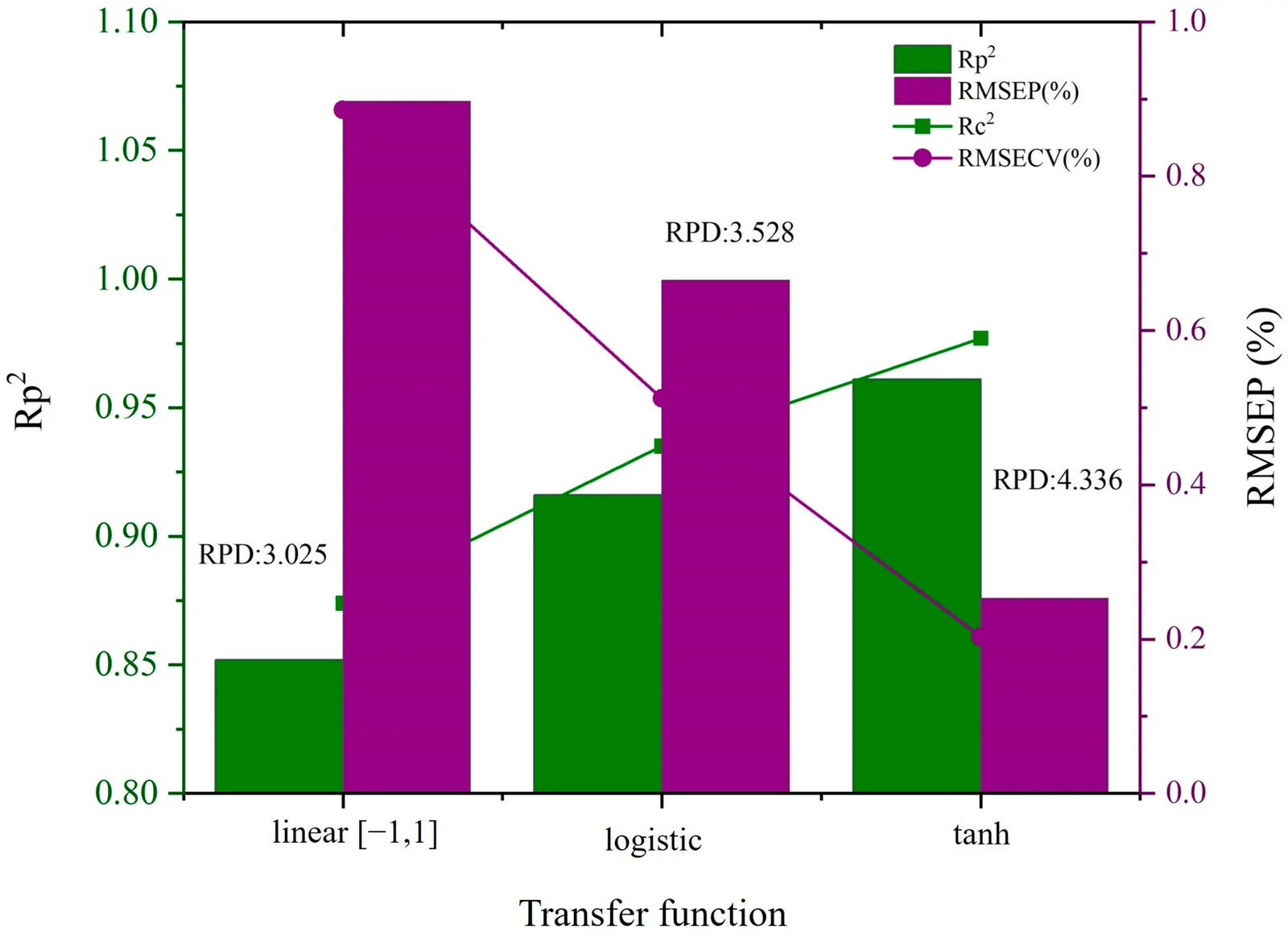
Comparison of prediction performance of BP-ANN models established using three different transfer functions.

Using the score vectors of the first three principal components (PC1–PC3) as network inputs and reference free amino acid (AA) contents as outputs, back-propagation artificial neural network (BP-ANN) models were established to quantitatively predict AA concentrations in Chongqing white tea. The Levenberg–Marquardt (LM) optimization algorithm was employed to update network weights and biases. Combining the empirical formula with grid search traversal, the number of neurons in the hidden layer was determined to be 6 based on the criterion of minimizing the RMSECV. The training hyperparameters were configured as follows: a maximum iteration limit of 1000 epochs, a learning rate of 0.1, and a convergence error goal of 0.001. Concurrently, an early stopping mechanism (with the maximum validation failure count set to 6) was implemented using the prediction set to dynamically monitor generalization errors and prevent model overfitting. Three distinct transfer functions—namely, the linear function (Linear [−1, 1]), the logistic sigmoid function (Logistic), and the hyperbolic tangent function (tanh)—were systematically evaluated, and the corresponding calibration and validation results are presented in Figure 6.

As illustrated in Figure 6, when the linear transfer function Linear [−1, 1] was applied, the determination coefficients of cross-validation and prediction were Rc^2^ = 0.874 and Rp^2^ = 0.852, respectively, accompanied by comparatively higher prediction errors (RMSECV = 0.886%, RMSEP = 0.897%) and an RPD of only 3.025, yielding the least satisfactory performance among the three functions. Incorporating the sigmoidal non-linear logistic function substantially elevated the goodness-of-fit, increasing Rp^2^ to 0.916, reducing RMSEP to 0.665%, and elevating RPD to 3.528. Notably, when the hyperbolic tangent function (tanh) was employed, the calibration model achieved optimal predictive performance: Rc^2^ and Rp^2^ reached 0.977 and 0.961, respectively; RMSECV and RMSEP dropped to 0.203% and 0.253%; and the RPD peaked at 4.336, demonstrating exceptional fitting precision and generalization robustness. This pronounced performance divergence originates from the intrinsic physical and chemical characteristics of tea spectroscopy. The near-infrared spectral profile of Chongqing white tea constitutes a continuous, complex multivariate signal characterized by strong overlaps of overtone and combination vibrational bands. Influenced by light scattering, complex matrix interferences, and multi-analyte absorbance superposition, an intrinsically non-linear relationship exists between spectral features and amino acid contents. The linear transfer function is constrained to linear feature space mapping, rendering it incapable of decoupling and modeling higher-order non-linear dynamics, which results in elevated residual errors. Although the logistic function introduces non-linear activation, its strictly positive output range of (0, 1) lacks zero-centered symmetry, which frequently induces gradient shifts during deep back-propagation, thereby impeding training efficiency and trapping weights in suboptimal local minima. In contrast, the tanh function provides a strictly zero-centered, symmetric response bounded within (−1, 1), exhibiting smooth gradient propagation that alleviates optimization oscillations and accelerates convergence. Consequently, tanh displayed superior sensitivity and stability in mapping complex non-linear spectral responses, enabling the BP-ANN model to achieve the highest predictive accuracy and analytical robustness [38].

#### 3.3.5. External Validation of Model Practical Performance

**Figure 7:**
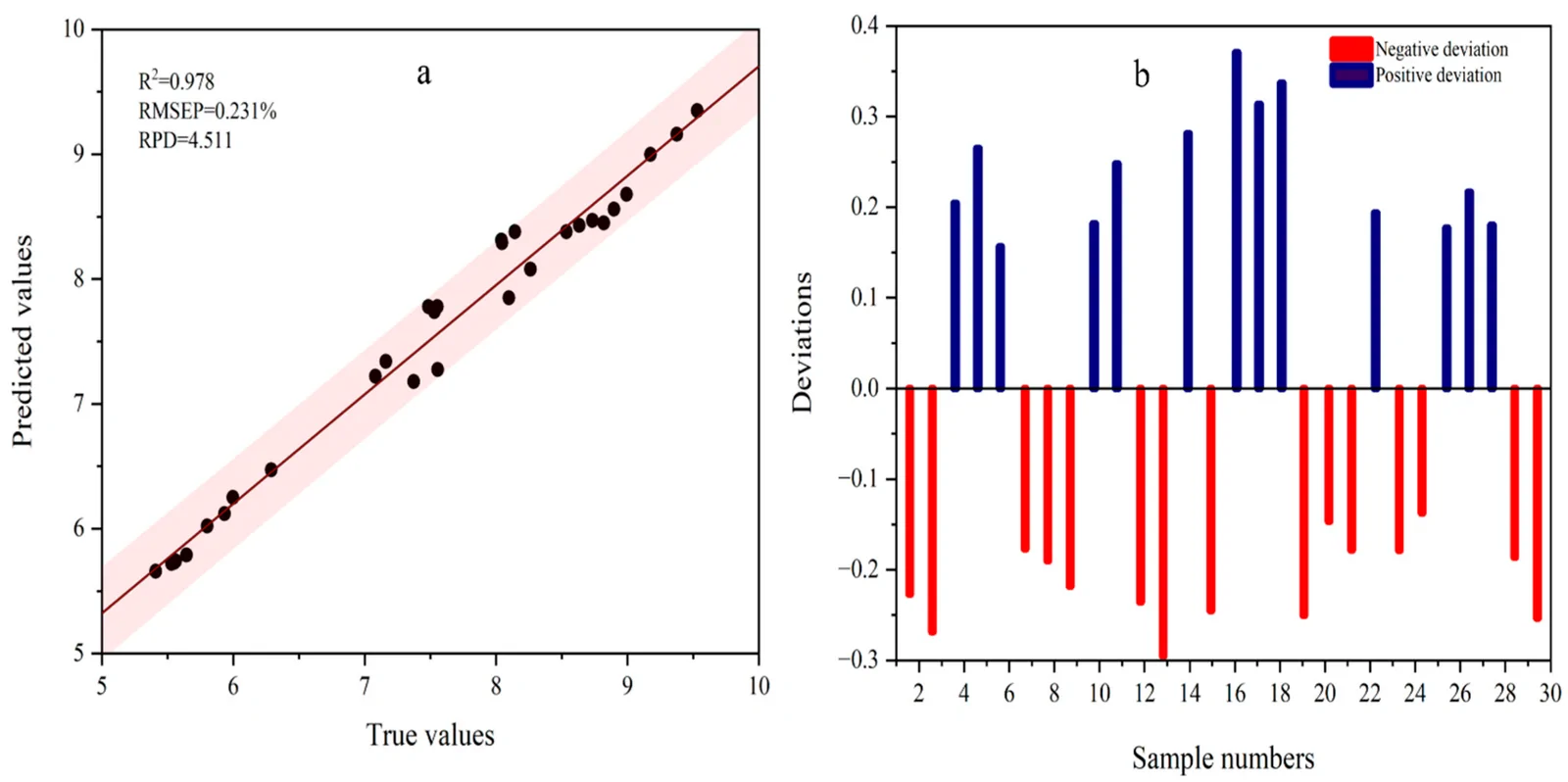
Validation and deviation analysis of 30 external samples for Chongqing white tea AA content by BP-ANN Model. (**a**) Scatter fit plot of predicted vs. true scores with R, RMSEP, and RPD metrics; (**b**) sample-specific deviations distribution.

To further verify the extrapolation generalization capability and predictive stability of the developed BP-ANN model in practical screening applications, an external validation set comprising 30 independent white tea samples not involved in calibration was predicted for AA concentrations. The results are depicted in Figure 7.

As demonstrated in Figure 7a, the reference AA concentrations of the external validation samples spanned from 5.41% to 9.53%, adequately covering the full dynamic range across low-, medium-, and high-quality tiers of white tea. The predicted values exhibited high consistency with the reference measured values, tracking the chemical variation trend tightly across all concentration gradients without perceptible bias or distortion. The absolute prediction deviations of all 30 external samples ranged from 0.14% to 0.37%, with all sample errors strictly constrained within 0.40%, and over 85% of samples displaying absolute deviations below 0.30%, demonstrating low overall relative prediction errors. Furthermore, across both low- and high-concentration intervals, the prediction deviations remained stably centered at approximately 0.15–0.25%, indicating that the network maintained balanced non-linear fitting and robust generalization across the entire calibration domain without boundary saturation distortion. Regarding the directional distribution of prediction residuals (Figure 7b), 13 samples exhibited positive residuals and 17 samples exhibited negative residuals across the validation set. Positive and negative deviations alternated randomly with comparable magnitudes, presenting no unilateral systematic overestimation or underestimation tendencies. This behavior substantiates the statistical unbiasedness and noise-resistant robustness of the model.

In summary, the external independent validation confirmed that the quantitative inversion model established via the optimized MSC + FD–si-PLS–PCA–BP-ANN framework achieved high fitting precision on the calibration set while delivering exceptional predictive reliability and analytical robustness for unknown blind samples, satisfying the technical criteria for rapid, non-destructive, and high-throughput quantification of free amino acids in white tea.

## 4. Conclusions

In this study, Chongqing white tea was investigated by integrating conventional wet chemical analyses, principal component analysis, and correlation analysis to uncover the core biochemical constituents dictating tea sensory quality. NIRS was subsequently coupled with linear PLSR and non-linear BP-ANN modeling to establish a rapid, accurate, and non-destructive digital quantification strategy. This work addresses the research gap in non-destructive spectral evaluation for Chongqing white tea and provides a reliable technical foundation and theoretical framework for intelligent quality grading and standardized industrial quality control. The principal findings are summarized as follows.

(1) Elucidation of biochemical profiles and identification of key quality markers.

PCA revealed that the first five principal components accounted for a cumulative variance contribution rate of 90.957%, comprehensively capturing the internal chemical variations among samples. PC1, PC2, and PC3 characterized bitterness and astringency, freshness and mellowness, and mellow sweet-aftertaste dimensions, respectively, which jointly governed the flavor formation of Chongqing white tea. In conjunction with correlation analysis, five pivotal quality-associated constituents were pinpointed: water extracts, free amino acids, gallic acid, EGCG, and caffeine. Among them, free amino acids served as the determining indicator governing the fresh and brisk flavor attributes, consolidating the biochemical foundation of Chongqing white tea characterized by “high amino acid content and a low polyphenol-to-amino acid ratio.”

(2) Optimization of spectral preprocessing and identification of characteristic spectral intervals.

Through the comparative evaluation of nine spectral preprocessing algorithms, the combination of multiplicative scatter correction and first derivative (MSC + FD) demonstrated the best performance in correcting baseline shift and scattering interference. Using synergy interval partial least squares (si-PLS), four characteristic subintervals ([2 3 7 10]) were isolated, effectively compressing the spectral array to 25% of the original variables while preserving key vibrational information of amino acid functional groups (C–H, C=O, and N–H). Further PCA dimensionality reduction on these characteristic intervals revealed that the first three principal components captured 97.16% of the cumulative variance, establishing a concise, robust input vector for subsequent neural network modeling.

(3) Superior predictive capability of the optimized BP-ANN model over linear algorithms.

The BP-ANN model configured with the hyperbolic tangent (tanh) transfer function achieved the highest predictive performance among the evaluated activation functions, yielding Rc^2^ = 0.977 and RMSECV = 0.203% in the calibration set, and Rp^2^ = 0.961, RMSEP = 0.253%, and RPD = 4.336 in the prediction set, substantially outperforming linear PLSR models. Independent external validation using 30 blind samples produced an R^2^ of 0.978 and an RMSEP of 0.231%, demonstrating high fitting precision, unbiased residual distribution, and balanced generalization across varying concentration gradients. Consequently, the established MSC + FD–si-PLS–PCA–BP-ANN (tanh) analytical pipeline enables rapid, non-destructive, and high-precision quantification of free amino acids in Chongqing white tea.

Although this study successfully developed a robust near-infrared prediction model for free amino acid contents in Chongqing white tea, several limitations remain that warrant further refinement from multidimensional perspectives: (1) Expanding sample diversity to enhance model generalizability. The sampling timeframe, geographical origins, and quality tiers investigated in this work were relatively restricted. Future research should incorporate tea samples spanning diverse plucking seasons, microclimatic cultivation environments, storage durations, and broader commercial quality grades. Expanding the representative sample spectral library will facilitate ongoing parameter recalibration and reinforce model robustness across variable real-world application scenarios. (2) Integrating multidimensional environmental and processing parameters. The present calibration framework was constructed solely on endogenous chemical profiles and vibrational spectral features, omitting exogenous variables such as manufacturing processing parameters, pedoclimatic conditions, and agronomic field management. Future investigations could assimilate multi-source heterogeneous parameters into multi-factor fusion models to capture subtle matrix fluctuations and further improve prediction precision. (3) Broadening machine learning algorithms and target quality attributes. Advanced chemometric and deep learning architectures—such as random forest (RF), support vector machines (SVMs), and convolutional neural networks (CNNs)—as well as ensemble learning frameworks, should be systematically explored. Concurrently, the quantitative calibration pipeline can be extended to simultaneously predict other pivotal quality indices (e.g., individual catechin monomers, caffeine, total soluble sugars, and water extracts), thereby establishing a comprehensive digital sensing platform for white tea quality evaluation. (4) Extending research dimensions toward functional bioactivities. The present study focused primarily on physicochemical quality and rapid optical screening. Subsequent work could combine in vitro and in vivo bioassays to elucidate the mechanistic correlations between the core chemical fingerprints of Chongqing white tea and its health-promoting properties (e.g., antioxidant and anti-inflammatory activities). This will facilitate the formulation of an integrative quality evaluation framework harmonizing sensory profiles, compositional metrics, and functional health attributes.

## Figures and Tables

**Table 1:** Descriptive statistics of sensory quality scores and biochemical component contents in Chongqing white tea.

Index	Max	Min	Average	SD
WE/%	48.65	42.10	44.25	2.43
TP/%	12.35	5.42	5.68	1.91
AA/%	9.66	5.41	6.56	1.36
TS/%	3.85	1.88	2.23	0.67
TC/%	11.53	9.18	10.64	0.62
GA/%	0.11	0.04	0.07	0.02
GC/%	0.48	0.12	0.28	0.08
EGC/%	2.18	1.30	1.82	0.73
C/%	0.20	0.10	0.13	0.06
EGCG/%	8.25	4.23	6.26	1.14
CAF/%	3.67	2.65	3.18	0.28
EC/%	0.60	0.36	0.46	0.07
GCG/%	0.48	0.21	0.35	0.08
ECG/%	2.68	1.55	2.23	0.52
Score	95.70	87.60	90.72	1.51

Note: Max: maximum; Min: minimum; SD: standard deviation; WE: water extract; TP: tea polyphenol; AA: amino acid; TS: total sugar; TC: total catechins; GA: gallic acid; GC: gallocatechin; EGC: epigallocatechin; C: catechin; EGCG: epigallocatechin gallate; CAF: caffeine; EC: epicatechin; GCG: gallocatechin gallate; ECG: epicatechin gallate.

**Table 2:** Eigenvectors and variance contribution rates of principal components.

Index	Eigenvectors				
	PC1	PC2	PC3	PC4	PC5
WE	−0.146	0.184	−0.089	−0.100	−0.280
TP	0.147	−0.256	−0.092	0.294	0.091
AA	−0.164	0.283	0.114	−0.290	−0.016
TS	−0.097	−0.102	0.287	0.235	0.266
TC	−0.038	0.119	0.275	0.481	−0.098
GA	0.231	0.223	−0.131	0.168	0.198
GC	−0.109	0.114	0.353	0.110	0.252
EGC	0.199	−0.102	0.335	−0.231	−0.103
C	0.212	0.039	0.148	−0.209	0.230
EGCG	0.197	0.232	−0.185	−0.022	0.213
CAF	0.141	0.279	0.014	0.215	−0.207
EC	0.158	0.076	0.319	−0.009	−0.356
GCG	0.117	−0.138	0.135	−0.285	−0.168
ECG	−0.035	0.075	0.103	−0.220	0.513
Initial eigenvalue	4.336	3.569	2.425	1.432	0.972
Contribution rates/%	30.971	25.493	17.321	10.229	6.943
Cumulative contribution rate/%	30.971	56.464	73.785	84.014	90.957

**Table 3:** Performance of calibration models based on characteristic spectral intervals selected by si-PLS.

Intervals	LVs	Selected Intervals	RMSECV (%)	Rc^2^
12	8	[4 8 9 12]	2.331	0.611
13	8	[4 8 10 12]	2.112	0.626
14	9	[4 5 10 14]	1.934	0.675
15	8	[2 9 12 14]	1.663	0.724
**16**	**6**	**[2 3 7 10]**	**1.225**	**0.812**
17	9	[9 14 16 17]	1.544	0.767
18	9	[5 7 17 18]	1.836	0.720
19	10	[5 14 18 19]	1.986	0.659
20	9	[10 18 19 20]	2.223	0.643
21	11	[5 8 12 20]	2.357	0.610
22	10	[12 18 21 22]	2.432	0.608
23	9	[8 18 22 23]	2.454	0.601
24	11	[10 14 23 24]	2.158	0.622
25	12	[20 21 24 25]	2.343	0.600

Note: Intervals, total number of partitioned equidistant subintervals; LVs, number of latent variables; Selected Intervals, optimal combination of subintervals selected by si-PLS; RMSECV, root mean square error of cross-validation; Rc^2^, determination coefficient of cross-validation. The optimal interval combination is highlighted in bold.

## Data Availability

The original contributions presented in the study are included in the article, further inquiries can be directed to the corresponding author.
